# Influence of Mastication on Orthodontic Wire Sliding in Nickel–Titanium Brackets with Superelastic Wings

**DOI:** 10.3390/jfb17080406

**Published:** 2026-08-15

**Authors:** Jasmin Wichelhaus, Uwe Baumert, Corinna Seidel, Hisham Sabbagh

**Affiliations:** Department of Orthodontics and Dentofacial Orthopedics, University Hospital, LMU Munich, Goethestrasse 70, 80336 Munich, Germany; jasmin.wichelhaus@med.uni-muenchen.de (J.W.); uwe.baumert@med.uni-muenchen.de (U.B.); corinna.seidel@med.uni-muenchen.de (C.S.)

**Keywords:** orthodontics, mastication, superelastic bracket, sliding mechanics, friction, binding

## Abstract

(1) Background: Orthodontic tooth movement (OTM) during sliding mechanics is influenced by a multitude of interactions. Studies suggest that vibration induced by mastication or usage of electric tooth brushes might impact resistance to sliding ($R_{S}$) in conventional brackets. In superelastic brackets, archwire sliding might also be altered due to deformation of the bracket wings during mastication. Therefore, the aim of this study was to investigate how the superelastic properties of Nickel–Titanium (NiTi) brackets affect archwire sliding during mastication. (2) Methods: Orthodontic superelastic NiTi archwires with diameters of 0.014” and 0.016” were ligated into four superelastic NiTi brackets. One bracket was positioned with a vertical offset of 1 mm to simulate clinical vertical leveling. A weight force was applied to axially preload the archwire. The occlusal and axial forces affecting the movement of the archwires through the bracket were measured. (3) Results: Low chewing forces caused a measurable axial movement of the 0.014” wire. Increased chewing forces resulted in higher axial movements. The archwire diameter had an impact: 0.014” NiTi required lower masticatory forces to initiate wire motion as compared to the 0.016” NiTi. (4) Conclusions: The superelastic NiTi brackets examined showed that under simulated continuous dynamic masticatory loading, obstacles such as friction or binding could be overcome.

## 1. Introduction

The matter of friction remains highly important in orthodontic straight wire therapy. While as little friction as possible is desirable during the leveling phases to enable sliding of teeth along the archwire, there are other clinically relevant situations in which friction serves as an anchoring factor. In the final phases of gap closure or during the application of torque, for example, friction may be useful in transmitting forces and moments to the teeth [1,2]. However, during the leveling phase, the motion of brackets along archwires is essential and may be impeded by tooth misalignment resulting in undesirable contact forces between bracket slot and wire. It is important to understand that orthodontic tooth movement (OTM) in general only takes place if friction between bracket and slot can be overcome [1,3,4]. 

Besides the friction controlled by normal force, the terms binding and notching play an important role for the effective OTM. Frictional force based on binding ($F_{BI}$) refers to an elastic deformation of the arch wire due to contact with the edges of the bracket slot. This is significant during the first leveling phases of OTM and leads to an increase in contact force between slot edges and archwire. In consequence, binding requires higher forces as compared to friction based on normal forces to induce movement. If binding occurs, the effect of elastic edge deformation dominates over the amount of friction generated by pure normal forces [5,6,7,8,9]. Notching ($F_{NO}$) is an increase in binding and leads to excessive forces at the contact points between bracket slot and wire due to plastic deformation of the archwire. Relative motion between wire and bracket is fully suppressed [10]. 

According to Kusy and Whitley [7], the following equation describes resistance to sliding ($R_{S}$) and frictional behavior between bracket and slot under the influence of binding and notching (1):

(1) $$R_{S}=F_{R}+F_{BI}+F_{NO}$$

$R_{S}$: resistance to sliding, $F_{R}$: friction based on normal force, $F_{BI}:$ frictional force caused by binding, $F_{NO}$: frictional force caused by notching.

The superelastic Nickel–Titanium (NiTi) brackets in this study were first described in 2017 [11]. The brackets are entirely made from superelastic Nickel–Titanium with a modified slot geometry, i.e., a V-shaped slot. This kind of superelastic material benefits from the constant environmental temperature in the human body, so that the superelasticity of NiTi can be reliably used [12]. However, in orthodontic applications, intraoral temperature may vary during the intake of hot and cold food and beverage, thereby affecting the stress-induced martensitic transformation of NiTi alloys. Based on the equation of Clausius-Clapeyron [13], an increasing temperature in NiTi components leads to higher stress levels which could possibly contribute to increased contact forces between the two friction partners. Thus, wire motion may be delayed or even constrained at higher temperatures. Contrary to that, cooling has the opposite effect and may reduce the stiffness of NiTi archwires, when, for instance, ice cold drinks lead to some pain relief after recent orthodontic archwire activation [14]. Once the intraoral temperature returns to physiological levels, the expected variation of contact stresses recovers back to its initial values. This in return means that if the temperature is kept constant but the stress level is varied, e.g., during occurrence of masticatory forces, the same stress reduction may be observed due to the deformation of the superelastic bracket wings’ structure.

The impact and changes in masticatory forces during straight wire therapy have been studied in different fields of dentistry [15,16,17]. It has been shown that orthodontic treatment improves occlusal contacts leading to a more consistent distribution of masticatory forces on numerous teeth [15]. In addition to occlusal loading, friction at the bracket–archwire interface is affected by tribological parameters, including surface roughness, lubrication, and edge rounding. Furthermore, studies reported that externally applied vibrations, such as those generated during mastication or by electric toothbrushes, may modify frictional behavior and could potentially facilitate archwire sliding [17,18].

The effect of masticatory forces on the mobility of the archwire–bracket interface has not been investigated so far, neither in conventional nor in superelastic brackets. Masticatory forces were not considered a relevant factor in sliding mechanics, likely due to the limited elasticity of stainless-steel brackets. Wings of superelastic brackets are able to deform reversibly during deflection of the archwire, hereby changing friction behavior [19]. Masticatory forces might also lead to deformation of bracket wings and potentially impact archwire sliding. Therefore, the aim of the present study was measuring the impact of masticatory forces on wire movement, leading to a change from static to dynamic friction. Furthermore, the effect of different axial loads on the relative movement of the wire during mastication was measured. These experiments should give an indication whether there is an impact of masticatory forces on the release of binding forces in brackets with superelastic wings, thus facilitating wire motion with a possible beneficial impact on OTM [20].

## 2. Materials and Methods

**Figure 1:**
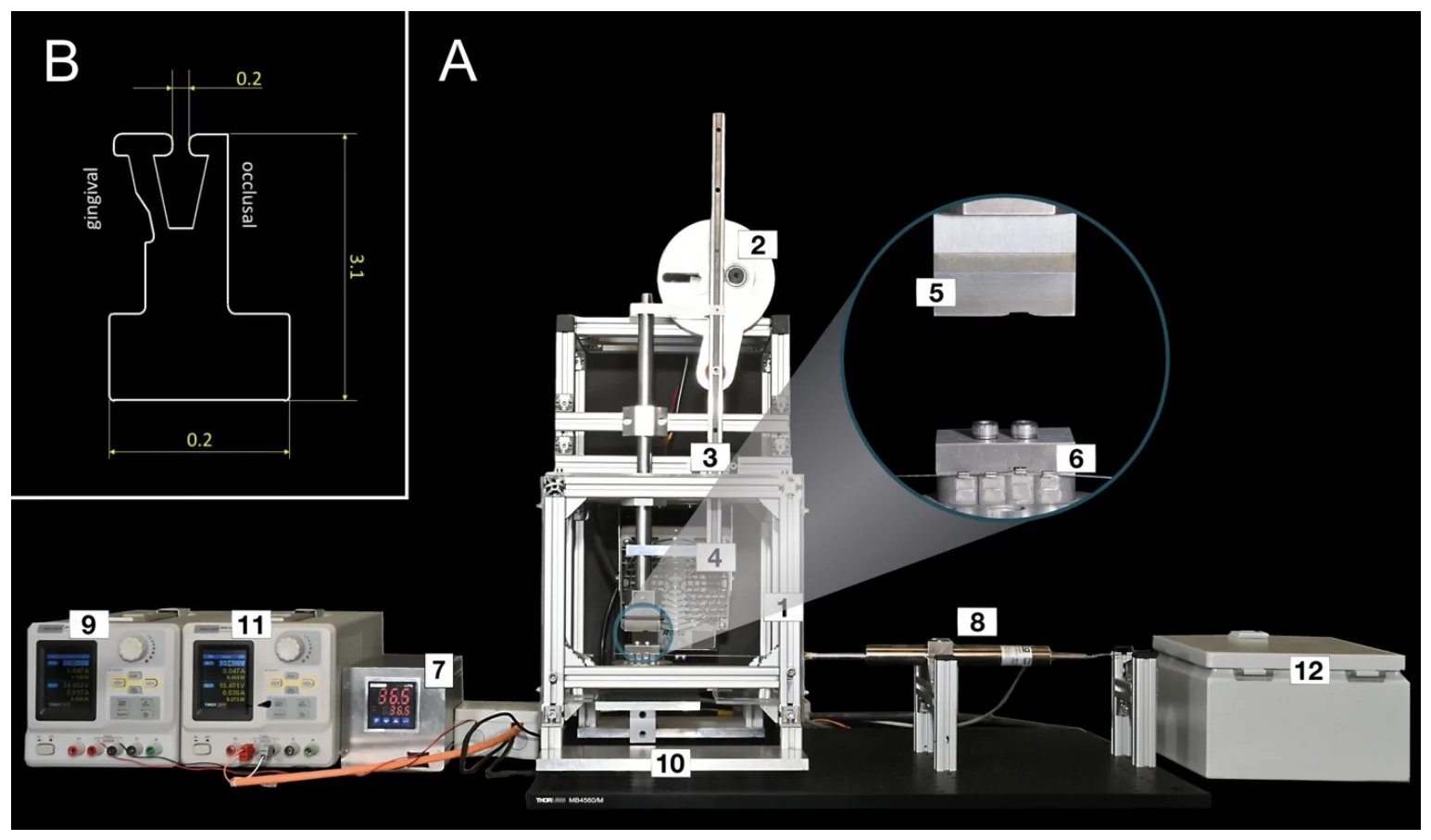
Overview over the chewing simulator and the modified RED bracket probes. (**A**) Chewing simulator overview and close-up view. (1) Frame made of aluminum profiles, (2) eccentric disc, (3) linear linkage, (4) heating fan, (5) plunger and (6) bracket holder, displaying four mounted brackets with one bracket 1mm intruded and a round 0.016” NiTi wire, (7) temperature controller, (8) displacement sensor, (9) displacement sensor power supply, (10) force sensor, (11) load cell power supply, (12) motor control unit. (**B**) CAD drawing of the modified RED bracket probes. The bracket base was modified from the clinical bracket base design to be securely fixed into the probe holder of the chewing simulator.

In this study, a chewing simulator was used in which four superelastic V-Slot brackets (RedSystem GmbH, Munich, Germany) were mounted, and cyclical forces were applied to the bracket wings in an occlusal direction to simulate chewing movements. A piston was vertically actuated by an eccentric disk and cyclically moved downwards in a linear motion, exerting a simulated chewing force onto the occlusal wings of the brackets (Figure 1A). In order to be able to fix the brackets securely in the chewing simulator, a modified bracket base design was manufactured for the testing procedure (Figure 1B). 

**Figure 2:**
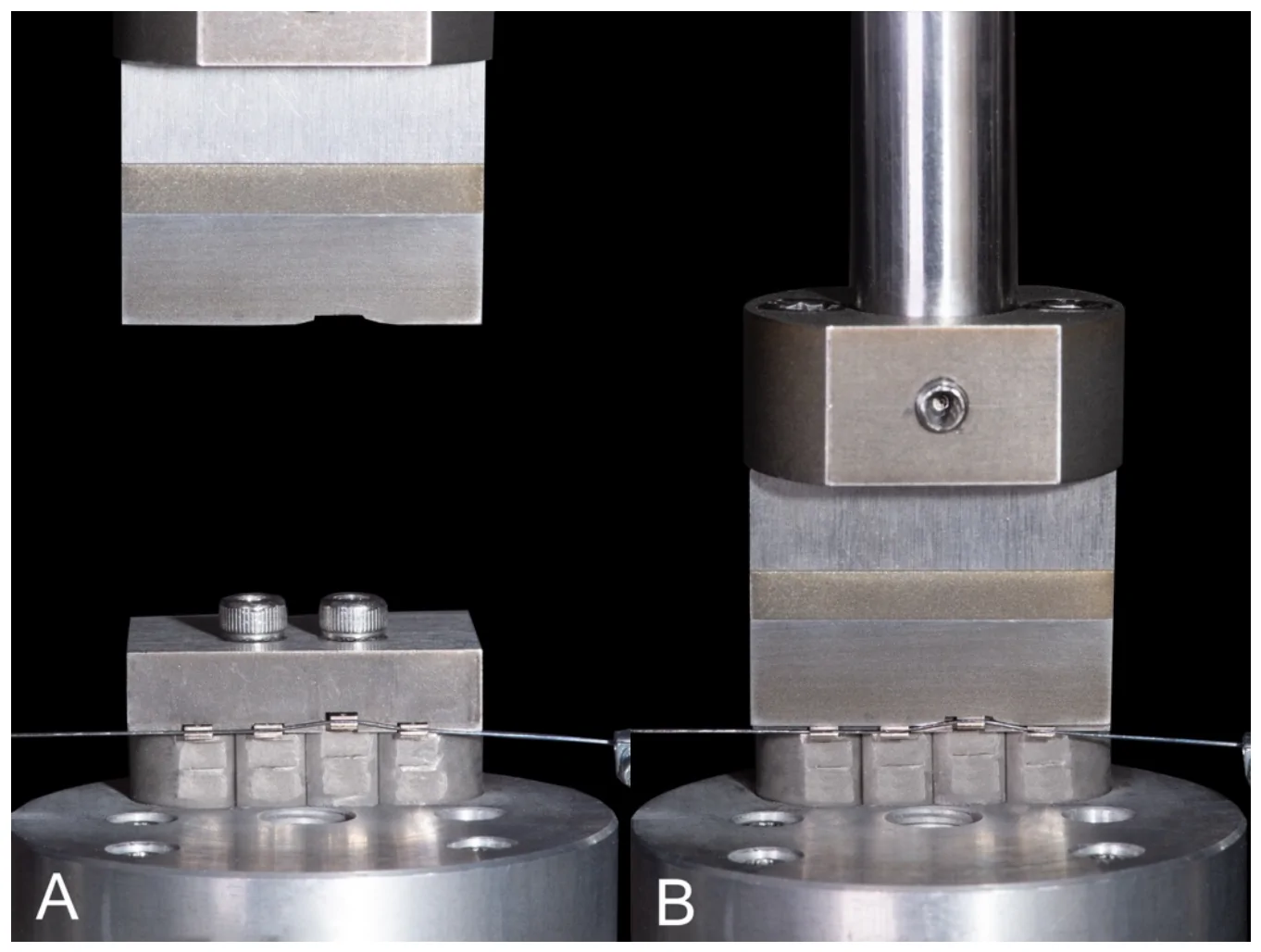
(**A**) Illustration of the plunger simulating the chewing movement during motion (**B**) and during exertion of chewing forces. The vertical offset of 1 mm of one bracket can be seen.

One of the brackets was mal-aligned and positioned 1mm off-center in occlusal direction in the setup to simulate an intruded tooth in the lower jaw, imitating the initial phases of orthodontic leveling and alignment (Figure 2).

As it was technically impossible to move the individual brackets along the archwire, it was decided to invert the principle of orthodontic tooth movement and move the wire, which was loaded with a small axial force through the slot of the fixed brackets. Relative motion between archwire and bracket remained the same. Hence, the experimental in vitro situation was an inversion of the clinical principle. Additionally, an inductive displacement sensor Inelta ISDL50-KD-2410 (Inelta Sensorsysteme GmbH & Co. KG, Taufkirchen, Germany) was added to the setup to measure the axial motion of the straight archwire. The ferromagnetic plunger of the Linear Variable Differential Transformer (LVDT) moved linearly through the coil, generating a proportional voltage. The distal end of the plunger was connected by means of a thin steel cable to a variable gravitational force using an idler pulley. This allowed the axial force application to be integrated into the test setup and the movement of the archwire became measurable. The LVDT calibration was carried out by using gauge blocks with 1.05 mm, 5 mm, and 10 mm (Hoffmann SE Group, Munich, Germany), so that the achieved sensor inaccuracy was about 1%. The calibration procedure was carried out multiple times during the course of the experiments. Due to the temperature dependence of the superelastic effect of both bracket material and archwire, a temperature chamber set to ϑ = 36.5 ± 1 °C was incorporated into the experimental setup. The temperature was maintained constant with a PID temperature controller EMKO ecoLITE 4.6.2R.0.0 (TDE Instruments GmbH, Steinenbronn, Germany).

The experiments included NiTi straight round wires with dimensions of 0.014” and 0.016” (Forestadent, Pforzheim, Germany), which were tested in previous biomechanical studies and are typically used in the orthodontic initial leveling phases [21]. The pre-set chewing force was varied between 20 and 100 N and was distributed equally to all brackets. The masticatory force data were recorded at a rate of 10 Hz using a calibrated load cell Siwarex WL260 SP-S AA (Siemens AG, Munich, Germany). Load cell calibration was carried out by use of calibration weights (Hoffmann SE Group, Munich, Germany) with an accuracy of 0.1% so that the final sensor inaccuracy was assumed to be 1% or better. Furthermore, the displacement weight varied between 0 and 105 g, corresponding to axial forces of 0–1 N. This value falls within the range of clinically relevant forces, potentially generated by orthodontic chains or coil springs [22]. Each measurement in this experiment was performed at least five times. A total of 250 chewing cycles were conducted with a frequency of 0.33 Hz in each experiment. This lower masticatory loading frequency was chosen to minimize possible strain rate associated effects during dynamic loading of the superelastic bracket material. Within each masticatory force group, the same archwire segment was used for five consecutive measurements. After each series of five measurements, the archwire was removed from the test rig, visually inspected for straightness and surface intregrity, and in case of inspection failure, replaced. After completion of all measurements within one masticatory force group, a new archwire was used for the subsequent force group. The masticatory forces were applied according to a predefined sequence for each archwire diameter. For the 0.014” archwire, the applied force levels were 30, 40, and 70 N, whereas for the 0.016” archwire, the sequence was 30, 70, 20, 40, and 100 N. Table 1 shows the experimental parameters relevant for this study. Initially, three baseline measurements were performed to demonstrate that, in the absence of axial weight application, the displacement results fluctuated around the zero point. The software LabVIEW 2020 (National Instruments, Austin, TX, USA) was used for experiment control, data acquisition of chewing forces, and axial displacement.

### Statistical Analysis

Statistical procedures were carried out with IBM SPSS Statistics Version 31.0.0.0 (Mac) (IBM Corporation, Armonk, NY, USA). Deviation from the assumption of normal distribution was tested with the Shapiro–Wilk test for each subgroup. Due to the small sample size and the likely deviation from the normal distribution for some of the subgroups, the Kruskal–Wallis test implemented in SPSS was used to examine the effect of chewing force and axial force variations on the dependent variable of the wire movement. In line with these findings, descriptive statistics of relative displacement were reported as median and interquartile range (IQR) for each wire dimension, axial force, and chewing force combination. To help readers understand the post hoc power analysis described below, the complete descriptive statistics, including mean and standard deviation, were reported in Supplementary Table S1. All test procedures were two-tailed considered *p* < 0.05 significant; for pair-wise post hoc tests, the *p*-value was adjusted using Bonferroni correction (details in Supplementary Table S2).

An a priori power analysis was considered but not conducted, since comparable data from published studies were not available to calculate effect sizes. Instead, post hoc power analysis for significant (*p*_Adj._ < 0.05) pairwise comparisons was carried out with G*Power software (version 3.1.9.6, macOS) [23] using the following settings: test family: ‘*t* tests’; statistical test: ‘means: Wilcoxon–Mann–Whitney test (two groups)´ or ‘means: Wilcoxon signed-rank test (one sample case)’; type of power analysis: ‘post hoc: compute achieved power’; two tails, and α error probability of 0.05. The effect size (Cohen’s *d*) and the corresponding power were extracted and reported (details in Supplementary Table S3 and Supplementary Figure S1).

In addition to the primary non-parametric analyses, a linear mixed-effects model (LMM) was established to account for the repeated-measures structure of the data. Relative displacement (mm) (continuous) was considered as the response variable, and wire dimension (nominal; 1 = 0.014”; 2 = 0.016”), axial force (ordinal; 0.4 N; 0.6 N; 0.7 N; 0.8 N; 0.9 N; 1.0 N), and bite force (ordinal; 20 N; 30 N; 40 N; 70 N; 100 N) as explanatory variables. To determine the individual random variability, wire ID (nominal; 50 levels, 11…115) was included as random intercept. The explanatory variables and interactions between them were added to the model (estimated using Maximum Likelihood (ML) and nloptwrap optimizer), and their contribution to the model was estimated using likelihood ratio tests and Akaike information criterion. The final model was estimated using Restricted Maximum Likelihood (REML) and nloptwrap optimizer, and contained wire dimension, axial force, and masticatory force as explanatory variables. Standardized parameters were obtained by fitting the model on a standardized version of the dataset. Confidence intervals (CIs) values and *p*-values were computed using a Wald t distribution with Kenward–Roger approximation. Model fitting was carried out using R (version 4.5.2) [24], and the R packages lme4 (version 2.0.6) [25] and lmerTest (version 3.2.1) [26]. Model quality was assessed using the R package performance (version 0.17.1) [27]. Multiple comparisons of model factors and their effect on relative displacement were calculated with emmeans (version 2.0.4) [28]. Reports of the models were prepared with the aid of the R packages report (version 0.6.4) [29] and the performance package, sjPlot (version 2.9.0) [30], and the packages from the tidyverse (version 2.0.0) [31]. The detailed model output is provided in Supplementary Table S4 and Supplementary Figure S2.

## 3. Results

**Figure 3:**
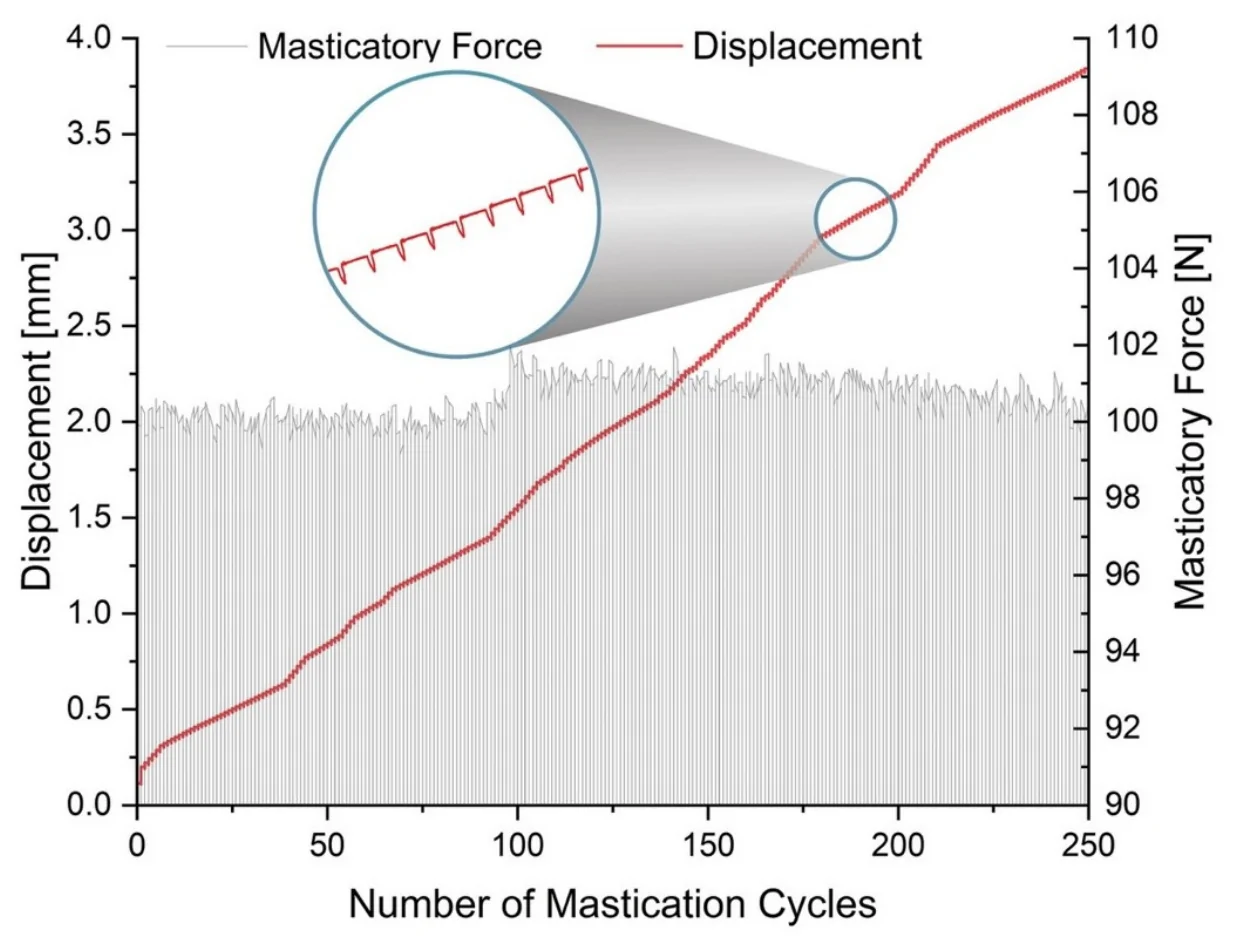
Example graph of a single test run. This trial displays a section from the experimental series using the 0.014” wire. The *x*-axis shows the number of mastication cycles per experiment, the *y*-axis displays the displacement of the wire in millimeters, and the *z*-axis shows the applied chewing force in Newton, fluctuating around 100 N in this case. The attached axial force was 0.6 N.

In all measured combinations of masticatory and axial forces, a wire movement was measurable during the applied load cycles. A typical graph showing the increase in wire displacement and the applied masticatory force over the number of cycles is shown in Figure 3. It was observed that both, the amount of masticatory force as well as the amount of axial force, affect the overall motion of the wire through the slot of the superelastic bracket investigated in this study during mastication. In the following section, the results were separated by archwire dimension, axial force, and chewing force. It was statistically analyzed whether there were differences between higher or lower chewing forces in different groups of axial forces.

**Figure 4:**
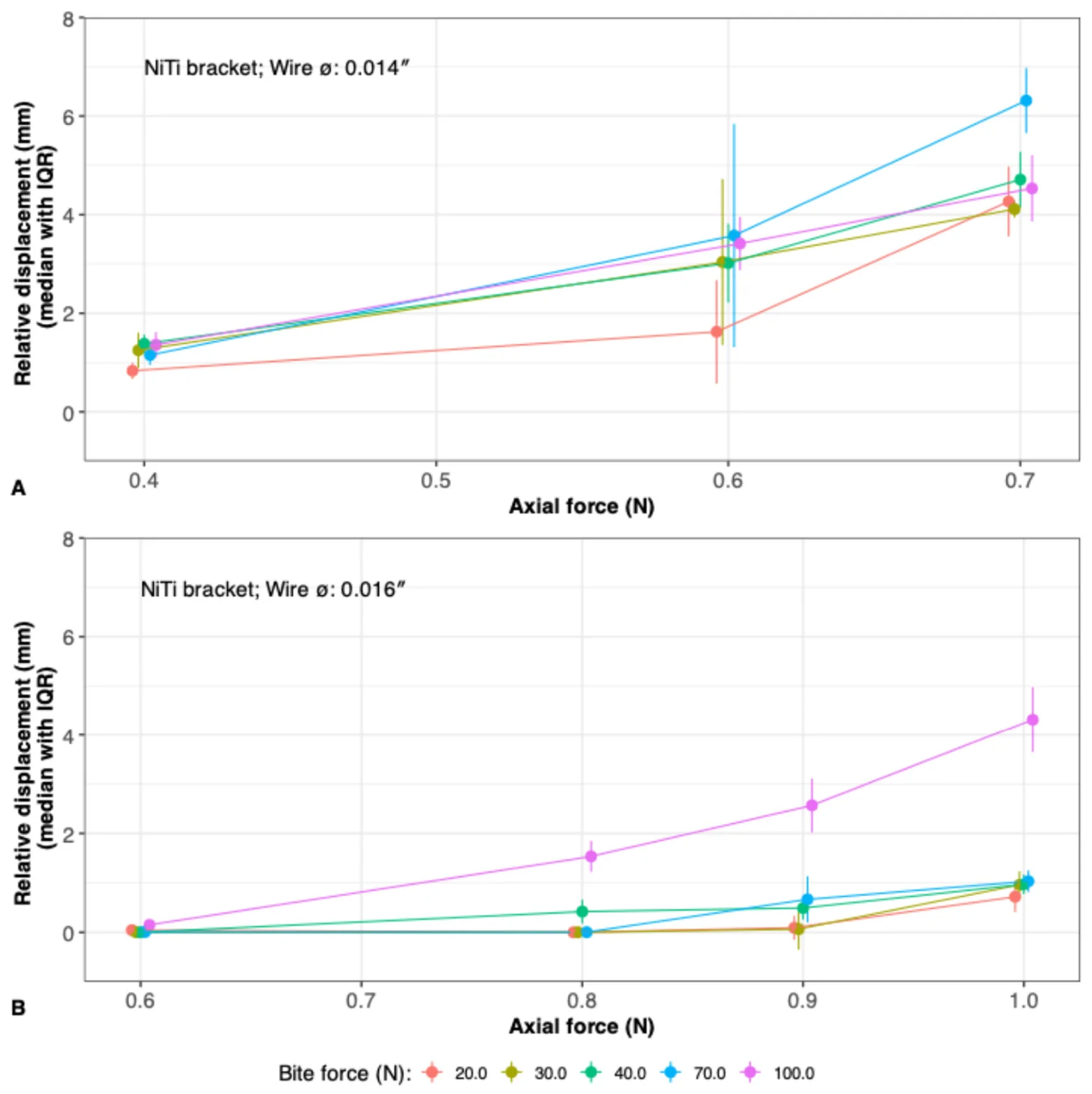
(**A**) Graphical representation of the experiments using the 0.014” wire as a function of the bite force and the different axial force groups. (**B**) The displacement of the 0.016” wire is shown in response to varying applied bite forces.

For visualization purposes, the median values of the relative displacement of the three axial force groups of the 0.014” wire (Figure 4A) and four axial force groups of the 0.016” wire were calculated and displayed in the following graphs (Figure 4B).

### 3.1. 0.014”. Archwire Diameter

The results of the 0.014” wire (Table 2) showed that minor displacements occurred at low chewing forces (20 N) in combination with low axial forces (0.4 N). The displacement increased with higher chewing forces at an axial force of 0.4 N from minimum 0.5 mm in the group of 20 N masticatory force to a maximum of 1.8 mm at a chewing force of 40 N. However, only a little wire motion at this range can be seen. Applying an axial force of 0.6 N resulted in a significantly higher overall wire displacement at higher chewing forces (70 N and 100 N) as compared to lower forces (20 N), reaching its maximum at 3.6 mm (*p*_Adj._ = 0.007; *p*_Adj._ = 0.002). The observed displacement difference corresponded to large effect sizes (Cohen’s *d* = 1.629; Cohen’s *d* = 2.630). Further increase in axial force to 0.7 N led to overall continued increases in wire displacement, reaching maximum values of 7.6 mm at 70 N. Significant differences were observed within the axial force range of 0.7 N and masticatory forces from 20 to 70 N (*p*_Adj._ = 0.009) with a large effect size (Cohen’s *d* = 3.252) and 30 to 70 N (*p*_Adj._ = 0.013). Smaller differences in masticatory forces (e.g., 40 to 70 N) led to insignificant results (*p*_Adj._ > 0.05) and were thus excluded from Supplementary Table S2. Although displacement seemed to increase with higher chewing forces, no significant difference (*p*_Adj._ = 1.00) was observed between 20 and 100 N at an axial force of 0.7 N. However, the observed effect size indicated a moderate effect (Cohen’s *d* = 0.460).

### 3.2. 0.016”. Archwire Diameter

In the group of the 0.016” NiTi wire (Table 2), the tests showed that with low chewing forces (20 to 40 N) and low axial forces (0.8 N), no wire displacement was measured. With higher chewing forces (100 N), slight wire movement commenced (maximum 0.4 mm) at low axial forces (0.6 N), showing a large effect size (Cohen’s *d* = 1). Further increase in axial force (0.9 N) led to a significant increase in wire movement from lower (20, 30 N) to higher chewing forces of 100 N (*p*_Adj._ < 0.001; *p*_Adj._ < 0.001), reaching maximum values of 3.9 mm of wire movement. This difference in wire movement was associated with a large effect size (Cohen’s *d* = 4.385). A similar pattern was found with the highest axial force (1 N); significant differences were observed between lower axial forces (20, 30, 40 N) and 100 N of chewing force (*p*_Adj._ < 0.001; *p*_Adj._ < 0.001; *p*_Adj._ = 0.005). Those significant differences in wire motion after Bonferroni correction were accompanied by large effect sizes (Cohen’s *d* = 7.242; Cohen’s *d* = 7.188; Cohen’s *d* = 7.4).

### 3.3. Linear Mixed Model

A linear mixed model was created to predict relative displacement at different axial force and bite force levels for each of the two wire dimensions. The model included the wire ID as a random effect. The model’s total explanatory power was substantial (conditional R^2^ = 0.826), and the part related to the fixed effects alone (marginal R^2^) was at 0.782. The model’s intercept (i.e., relative displacement [mm]), corresponding to a 0.014” wire, a bite force of 20 N, and an axial force of 0.4 N, was at 0.48 mm (95% CI, 0.07–0.90, *p* = 0.024) (Supplementary Table S4).

After adjusting for the other variables in the model, relative displacement decreased for 0.016” wires (β = −2.90 [95% CI, −3.29 to −2.50], *p* < 0.001) in comparison to the 0.014” wire dimension.

Bite force: after adjustment for the other variables in the model, relative displacement increased (β = 0.34 [95% CI, −0.10 to 0.79], *p* = 0.123) for 30 N compared with the reference. With further increasing bite force, the relative displacement increased gradually from 0.56 mm (95% CI: 0.12 to 0.99, *p* = 0.014) for 40 N bite force to 1.79 mm (95% CI: 1.35 to 2.22, *p* < 0.001) for 100 N bite force, after adjustment for the other variables in the model.

For each level of axial force, relative displacement increased on average by 1.76 mm (95% CI: 1.41 to 2.10) for 0.6 N axial force up to 3.35 mm (95% CI: 2.86 to 3.83) for 1 N axial force after adjustment of the other variables in the model. The effect of axial force was for all levels of axial force highly significant (*p* < 0.001).

Estimated marginal means of relative displacement across the different levels of bite force, based on the predictions from the LMM, are given in Supplementary Figure S2 for both wire dimensions. More complex models, i.e., models with interactions or nested groups as random factors, were tested but dismissed as all showed signs of overfitting.

## 4. Discussion

The influence of friction on orthodontic tooth movement (OTM) continues to be of great importance and is frequently compared in various studies between self-ligating brackets and conventional stainless-steel brackets [4,32,33,34,35,36,37]. Nevertheless, there are few studies which discuss the influence of friction within the bracket–wire interface. Articolo and Kusy [38] were able to prove in their experiments that with an angulation of 7° between bracket and wire, binding represented 80% of the resistance to sliding ($R_{S}$). An increased contact angle of 13° resulted in a binding influence of 99% of the overall ($R_{S}$) [10,38]. Articolo and Kusy et. al. [38] showed that the dominant effect in friction is based on binding at the bracket edges. Recent studies [39] confirmed this, as different contact configurations at the bracket–wire interface had a significant impact on resulting forces and moments, with more bracket–wire contacts the effective wire stiffness (EWS) increased, thus potentially hindering the sliding of the wire. Normal force-based friction can be regarded as a system-specific parameter of the bracket–archwire interface, which is being affected by numerous variables such as surface roughness, wire geometry, bracket edge rounding, lubrication, and the presence of food debris [40]. Consequently, normal force-induced friction had a minor impact on wire sliding and was not considered relevant in this study. Therefore, Formula (1) of Kusy [7] previously mentioned was adjusted as follows (2):

(2) $$R_{S}=F_{BI}+F_{NO}$$

**Figure 5:**
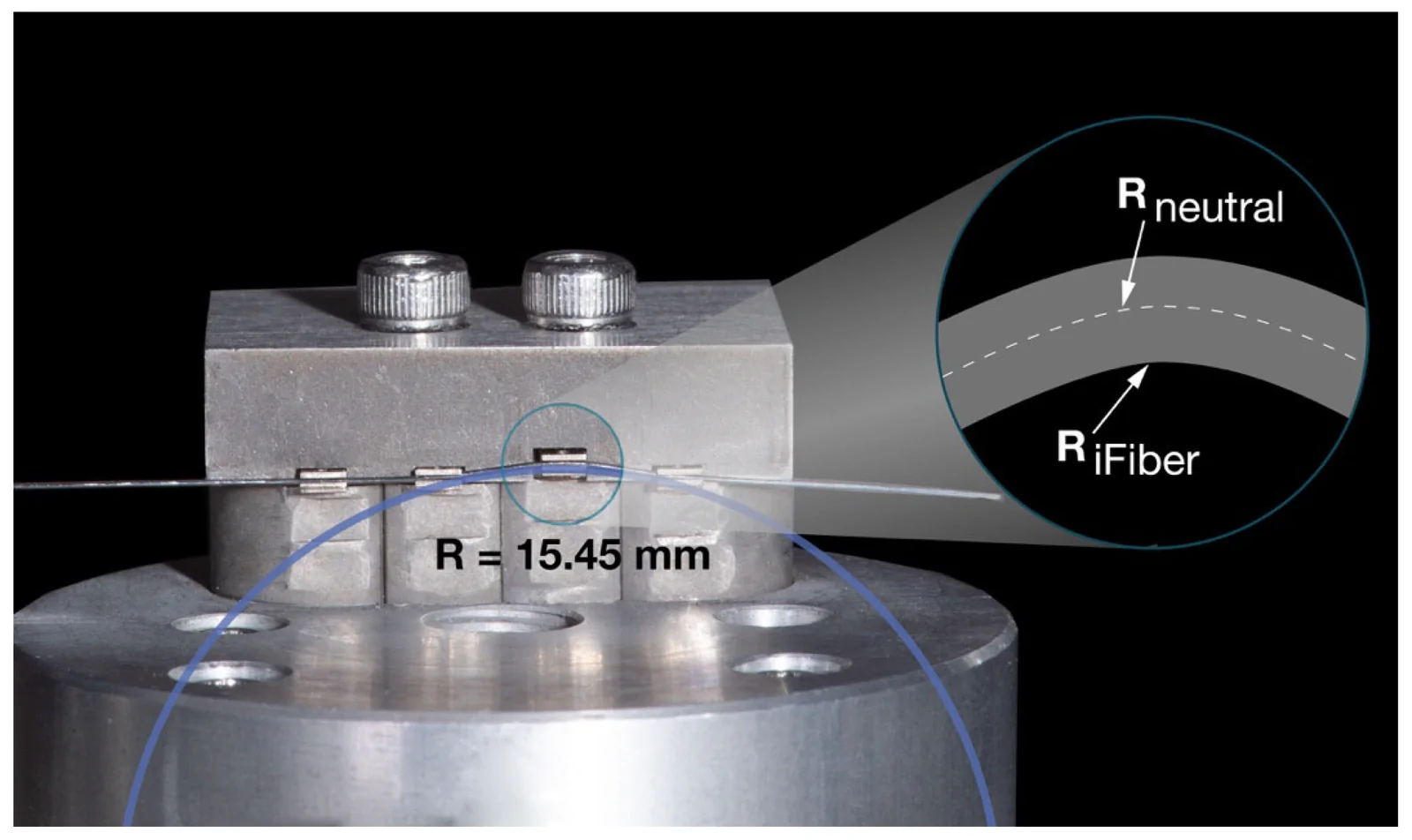
The close-up view displays the experimental setup with the 0.016” NiTi wire showing the edge fiber elongation for the inner fibers. The magnifying lens presents the conceptual geometrical arrangement of the fibers.

For the present study, Formula (2) can be further simplified as the elastic deformation and thus only the occurrence of binding can be confirmed and estimated by using inner edge fiber strain calculations based on the larger archwire dimension of 0.016” (Figure 5). The concentric radius of the inner fiber was determined using a magnified photograph of the experiment (Figure 5). To identify the radius of the neutral edge fiber, the following equation was applied (3):

(3) $$R_{neutral}=R_{iFiber}+\frac{d}{2}$$

$R_{neutral}$: radius of the neutral fiber, $R_{iFiber}$: radius of the inner fiber, d: wire diameter.

Smaller wire dimensions lead to reduced outer fiber strains, hence the risk of plastic deformation and the occurrence of notching is considerably lower. While the neutral fiber of a wire in bending mode does not experience any strains, the outer fiber is symmetrically elongated as compared to the inner fiber and the strain is calculated based on purely geometric consideration for linear elastic materials (4):

(4) $$\varepsilon =\frac{\Delta L}{L}=\frac{L_{neutral}-L_{iFiber}}{L_{neutral}}=\frac{15.651\;\mathrm{mm}-15.450\;\mathrm{mm}}{15.651\;\mathrm{mm}}\approx 0.013\;\mathrm{mm}=1.3\%$$

$L_{neutral}$: length of the neutral fiber; $L_{iFiber}$: length of the inner fiber.

With a calculated inner fiber strain of 1.3%, any superelastic NiTi wire is still well within its elastic strain regime, which ends at approximately 8% [41]. Therefore, a plastic deformation, which is the basis of notching, can be excluded and $F_{NO}$ can be neglected too.

Hence, $R_{S}$ was simplified to approximately equal $F_{BI}$, as it was assumed that under the investigated conditions, notching can be neglected and frictional resistance is essentially represented by binding. Kusy’s formula was reduced to the following (5):

(5) $$R_{S}=F_{BI}$$

However, this simplified formula should be interpreted as representing a controlled assessment of binding under standardized laboratory conditions rather than the full spectrum of R_S_ encountered clinically, where simultaneous friction, binding, and notching are expected to occur.

**Figure 6:**
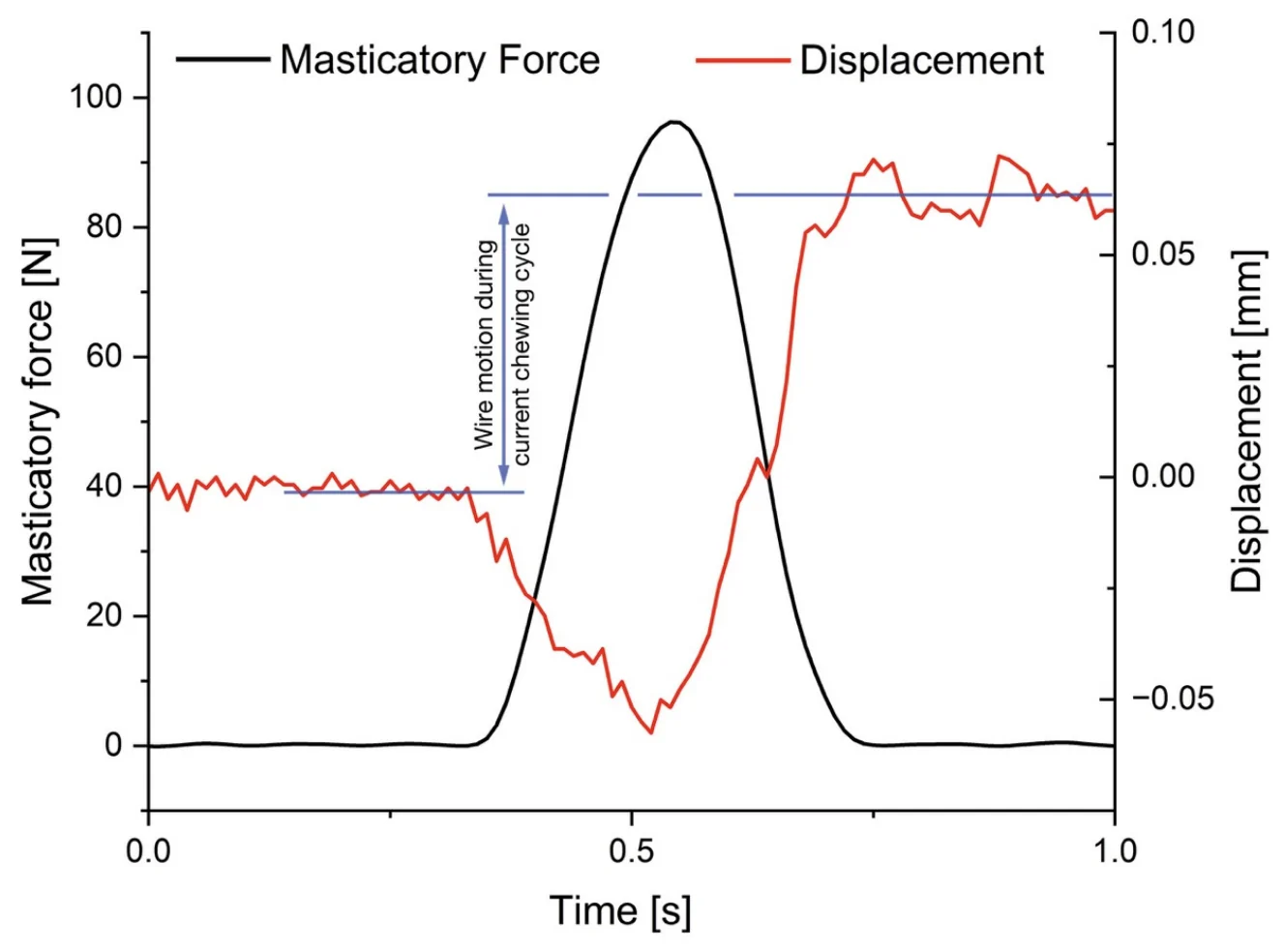
Single mastication cycle excerpted from an experimental measurement. Averaging the position before and after mastication contact resulted in a difference in wire position of approximately 0.06 mm. It clearly demonstrates that the wire is moving through the slot during mastication.

In many tribological systems, the transition from static to kinetic friction is unstable and is accompanied by the occurrence of the so called ‘stick and slip’ effect, whereby ‘stick’ describes the repetitive adherence of the surfaces in contact to each other due to dominance of static friction, followed by a release of frictional force during ‘slip’ towards kinetic friction [42]. The experiment in the present study is similar to this mechanism (magnification in Figure 3). The discontinuous movement of the wire out of the slot may be consistent with the occurrence of the stick–slip mechanism, as the wire ‘sticks’ and subsequently ‘slips’ during the impact of chewing [18]. A further magnification of the data from Figure 3 gives insight into the exact process of motion activation between wire and slot during mastication (Figure 6). Similarities to other studies [18] using ultrasonic vibrations such as toothbrushes can be identified. However, whilst the present study used low force frequency and high amplitude, the opposite holds true for electric toothbrushes.

### 4.1. 0.016”. Archwire Diameter

Further interesting aspects with the superelastic NiTi brackets showed that the movement of the 0.016” wire is dependent on the amount of masticatory as well as axial force (starting from 0.8 N) clinically induced by either a spring or an elastic chain. With the 0.016” wire, there were significant differences from lower to higher chewing forces in nearly all applied axial forces, with only the group of the lowest axial force of 0.6 N as an exception. Thus, leading to the assumption that a minimum amount of axial force is needed to achieve reliable movement of the 0.016” wire in the slot for all groups of chewing forces. By increasing the masticatory force and the axial force, the wire can release quicker from the edges of the bracket (Figure 3 and Figure 6).

### 4.2. 0.014”. Archwire Diameter

**Figure 7:**
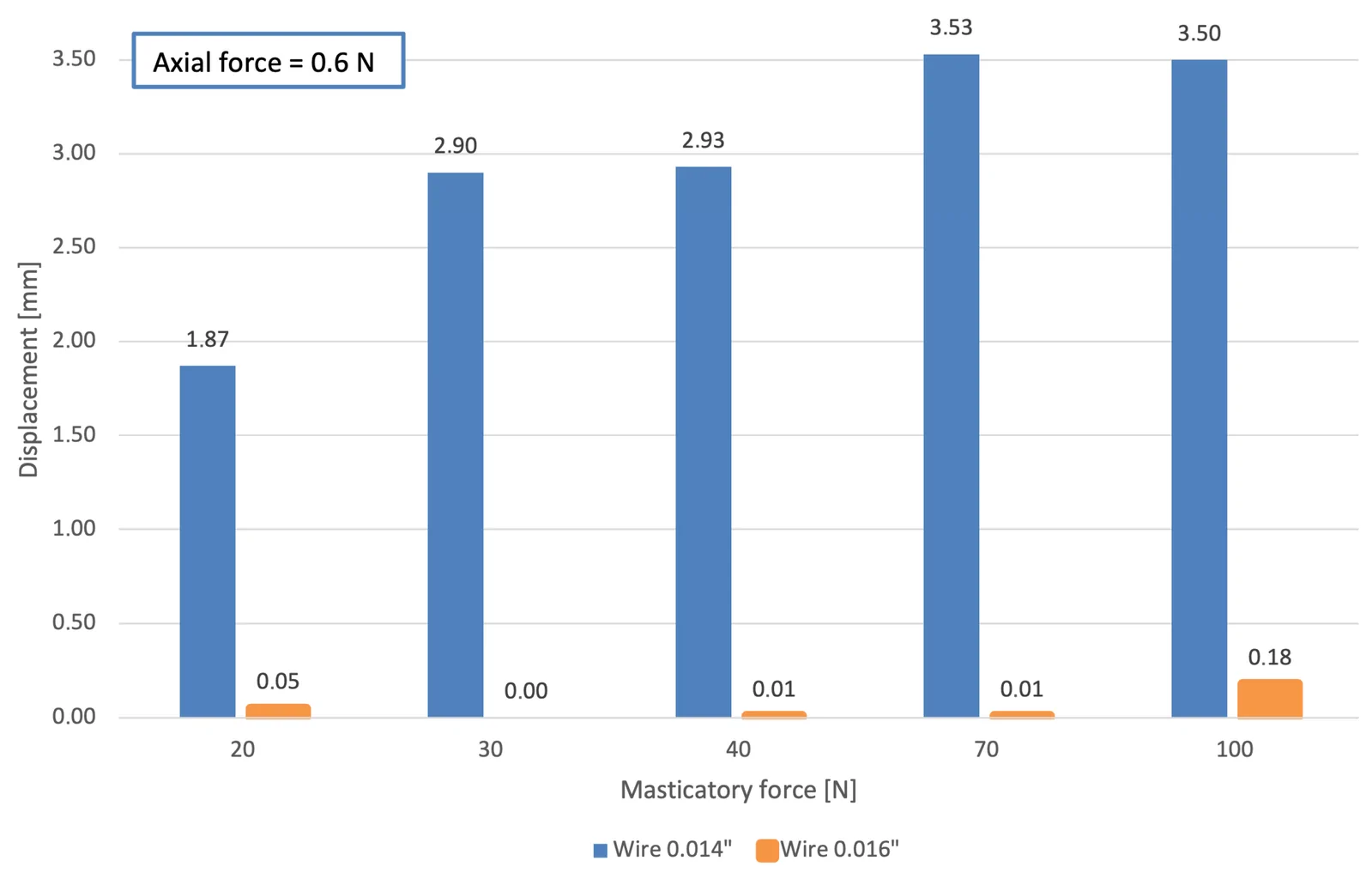
Illustration of the total displacement under constant axial force of 0.6 N in both archwire dimensions (0.016” and 0.014”) for all masticatory forces tested. The column thickness (orange) was adjusted for enhanced graphical visibility.

The archwire diameter was the most important variable. As the wire dimension increased (0.016”), frictional effects, most likely based on binding, dominated and the speed of movement of the wire through the slot decreased drastically per mastication cycle compared to the 0.014” archwire. Hence, the 0.014” wire permitted significantly more movement compared to the 0.016” wire with a given chewing and axial force, likely due to the larger wire-bracket play and the reduced contact forces at the edges of the bracket slot (Figure 7) [39]. Analyzing the results of the 0.014” wire, it became evident that the variation in chewing forces had no significant impact on overcoming binding forces and wire movement. Furthermore, in Figure 7, there appears to be a force limit above which higher chewing forces do not lead to an increase in wire motion. The confine value may be between 70 and 100 N of chewing force with the possible reason that the deformation of the superelastic bracket is so significant that further increase in masticatory forces does not contribute to a reduction of binding forces anymore. Therefore, it may be said that chewing forces did not directly impact wire motion most likely due to a larger play in the bracket slot and the limited applicable axial force range. Beyond the value of 0.7 N axial force, wire sliding movement occurred independent of chewing forces. Consequently, the chewing force was not the determining factor anymore for wire movement. Therefore, 0.7 N of axial force was defined as the upper testing limit.

Furthermore, experiments with the NiTi bracket and the round 0.014” wire showed that high axial forces, clinically generated by elastic chains or springs, were not required to induce wire movement. Rather low forces were sufficient and are considered more physiological in orthodontics [43,44]. Axial forces out of the biological range, on the other hand, can lead to tilting of teeth, larger hyalinization areas, and root resorptions [22,45,46,47]. If high forces are applied, a substantial amount of force is lost due to excessive binding or normal force-based friction forces which need to be overcome before OTM takes place. The elasticity of the bracket has no influence on these high force values.

### 4.3. Masticatory Forces

There are many studies that have measured direct occlusal forces in different areas between the surfaces. In the molar region, these forces vary from 100 up to 1000 N, depending on the patients’ gender and age [48,49,50,51,52]. However, the direct chewing forces do not impact on the bracket, which are rather exposed indirectly to shear forces of masticated food. To date, there have been no studies that have examined and can estimate the amount of force exposure to the bracket due to mastication without causing debonding or fracture of bracket wings. It can be concluded that these forces do not act on a single bracket, as the wire distributes the forces to multiple brackets. The upper limit of forces acting on brackets can be estimated based on the shear stress τ of common adhesives from the literature which is around 5–15 MPa [53,54,55,56]. This means that a maximum force on a V-slot bracket (area of the bracket base A = 7 mm^2^) can be calculated by (6):

(6) $$F_{max}=\tau \cdot A=15\;MPa\cdot 7\;{mm}^{2}=105\;N$$

Due to incorrectly performed bonding protocols, brackets can debond well before reaching this theoretical maximum load. Therefore, the chewing forces in this study have been determined to be in the range from 20 to 100 N. However, these hypothetical values are based on theoretical considerations, which may not directly represent the clinical forces transmitted to orthodontic brackets.

### 4.4. Deformation of Bracket Wings in Stainless-Steel Twin Brackets

Stainless-steel brackets were not part of the present in vitro study. Therefore, the following considerations explain the rationale for excluding stainless-steel brackets from the study.

**Figure 8:**
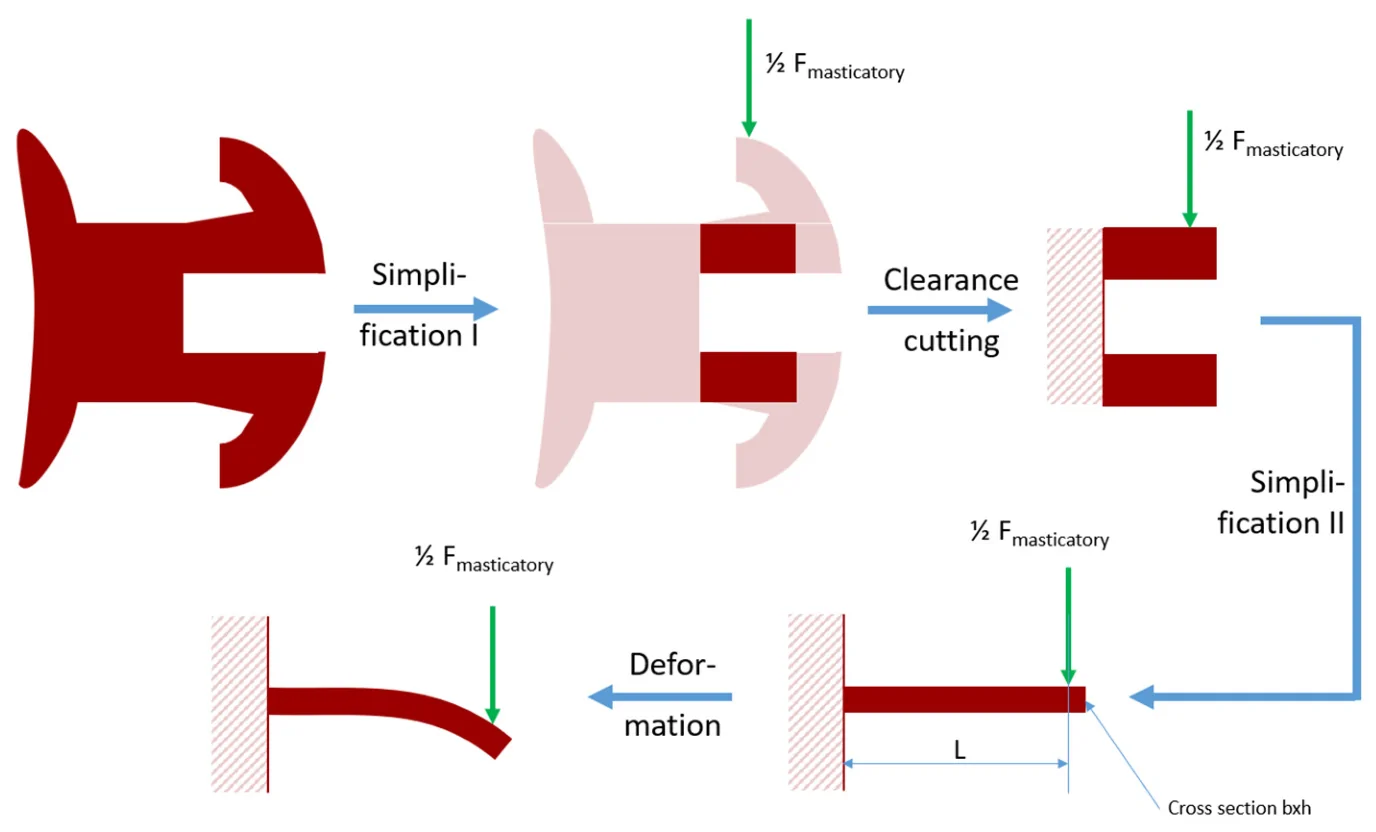
Illustration of possible bracket deformation under masticatory force (F_masticatory_) in a stainless-steel twin bracket. The calculation is based on a simplification of the design in which only one of the two wings is considered.

To calculate the possible deformation of the bracket wing on conventional stainless-steel Mini Sprint brackets (Forestadent, Pforzheim, Germany), it was simplified and assumed that the individual bracket wing can be considered a cantilever beam fixed at one end and subjected to a point load at the free end (Figure 8). This assumption is based on the fact that the connection between bracket wing and bracket base can be regarded as a statically rigid connection.

The maximum applied chewing force in this experiment of 100 N, which was evenly distributed to both bracket wings, results in a bending force of F_masticatory_ = 50 N. The dimensions of the bracket wing are 1.15 mm in length (*l*), 1.08 mm in width (*b*), and 0.44 mm in height (*h*). The resulting area moment of inertia (*I*) of the bracket wing is therefore as follows:

(7) $$I=\frac{bh^{3}}{12}=\frac{1.08\;mm\cdot 0.44^{3}\;{mm}^{3}}{12}=1.83997\cdot 10^{-3}\;{mm}^{4}$$

The Youngs’ modulus (E) for stainless steel is around 200.000 N/${mm}^{2}$. Hence, the deflection (*f*) under load (F) of the bracket wing with a measured length of 1.15 mm (*l*) can be estimated as follows [57]:

(8) $$f=\frac{F\cdot l^{3}}{3\cdot E\cdot I}=\frac{50\;N\cdot 1.15^{3}\;{mm}^{3}}{3\cdot 200.000\;N/{mm}^{2}\cdot 1.83997\cdot 10^{-3}\;{mm}^{4}}=0.069\;mm\approx 69\;\mu m$$

The estimated deflection of 69 µm at the distal end of the bracket wing leads to an outer fiber strain of the stainless-steel wing according to equation (4):

(9) $$\varepsilon =\frac{∆L}{L}=\frac{0.22\;mm}{9.9\;mm}\approx 0.022=2.22\%$$

The calculated deflection of 69 μm as well as the calculated strain of 2.22% when applying 100 N of force onto bracket wings of stainless steel suggests the occurrence of plastic deformation in stainless-steel brackets under the applied masticatory load [58]. Furthermore, the cyclic occurrence of high masticatory loads may lead to an accumulation of plastic deformation. It has thus been decided to relinquish on measurements of stainless-steel-based brackets.

### 4.5. FEM Simulations of the Aforementioned NiTi Brackets with Superelastic Wings from Previous Work

**Figure 9:**
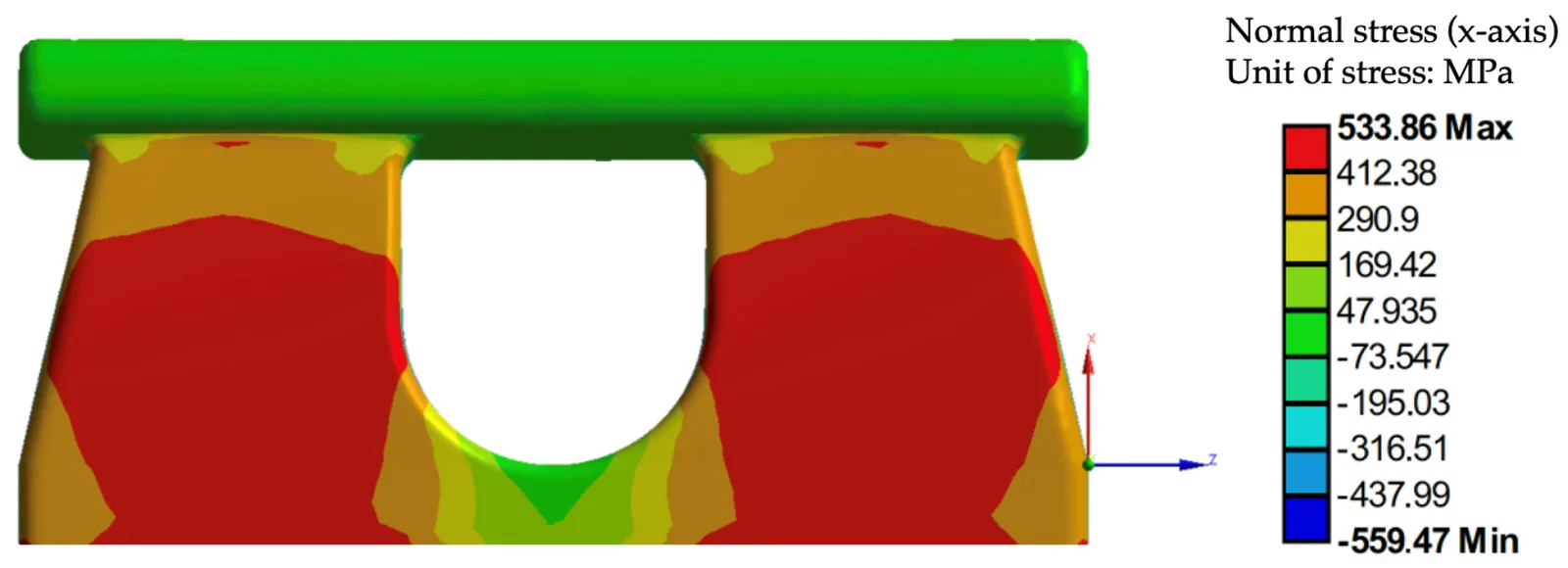
FEM simulation showing the relatively low and even normal stress distribution in the x-direction on the bracket wings. Reprinted with permission from Ref. [19].

**Figure 10:**
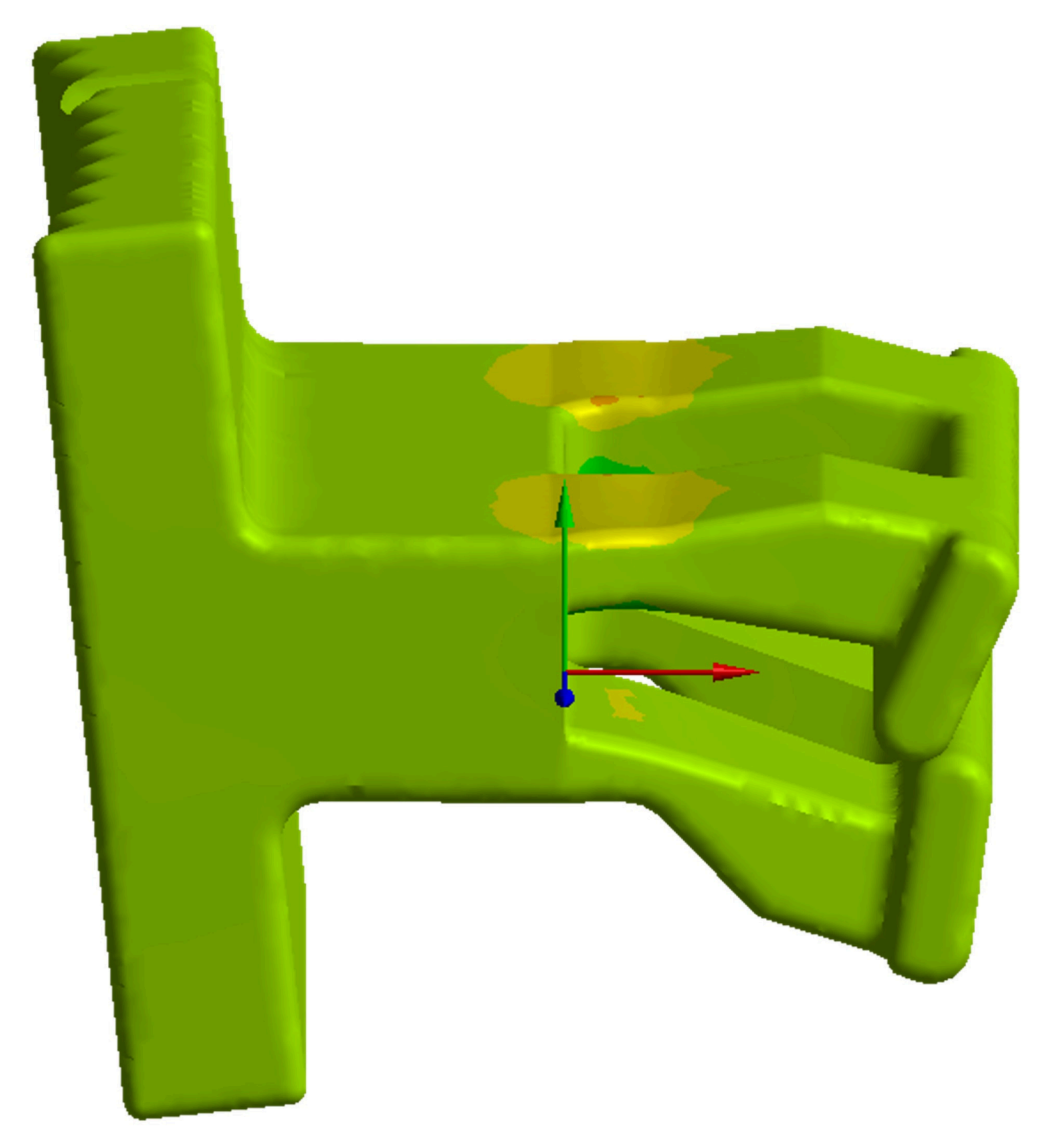
FEM simulation showing the deformation of a previous bracket design iteration under masticatory load. Reprinted with permission from Ref. [19].

Previous FEM work (Figure 9 and Figure 10) demonstrated that for the given bracket design equivalent, stresses below 560 MPa have been calculated [19]. Typical martensitic transformation stresses for nickel–titanium are in the range of 400 to 550 MPa [59] so that the occurrence of a superelastic transformation-based deformation can be assumed.

FEM simulations on previous iterations (Figure 10) of said superelastic bracket have shown that significant elastic deformation takes place upon the occurrence of masticatory forces [19]. This significant deformation illustrates that existent binding forces could well be released possibly leading to faster OTM and shorter orthodontic treatment duration, which needs to be verified by further clinical investigations.

### 4.6. Vibration Induced by Mastication

Previous studies have shown that vibrational stimulation and masticatory forces may influence orthodontic biomechanical behavior [60,61,62,63,64,65]. However, direct comparison with the present findings is limited due to differences in experimental approaches. Regarding masticatory forces, previous investigations demonstrated that occlusal loading, simulated by chewing gum, affected frictional behavior during sliding mechanics but did not eliminate frictional resistance [66]. Nevertheless, this study investigated conventional sliding mechanics using stainless-steel brackets and stainless-steel archwires [66], whereas the present study focused on specifically described superelastic brackets and NiTi archwires during the leveling and alignment phase. Studies investigating vibrational stimulation have mainly evaluated its possible effect on the rate of OTM rather than its influence on $R_{S}$ [60,61,62,63,64,65]. While most prospective randomized studies reported no clinically relevant acceleration of OTM during alignment or canine retraction using low-frequency vibration devices [60,62,63], one study reported an increased rate of OTM [61]. Taken together, current evidence indicates that dynamic mechanical factors may influence OTM; however, their clinical relevance remains inconclusive. The present findings should therefore be interpreted within the context of the investigated bracket–wire system and loading conditions.

### 4.7. Limitations and Strengths

With regard to the experimental setup, several aspects should be considered when interpreting the findings. First, only a single bracket design in combination with round NiTi archwires was investigated. This configuration was intentionally selected as the investigated bracket is currently the only commercially available superelastic bracket featuring superelastic bracket wings and a trapezoidal slot geometry [11]. Hence, comparison with conventional stainless-steel brackets was not possible, as these differ not only in the material properties of the bracket wings but also in slot geometry. In addition, the force delivery of NiTi archwires is strongly influenced by bracket-slot geometry. Smaller slot dimensions (e.g., 0.018” versus 0.020” slots) have been shown to generate higher force levels [67]. Therefore, comparison with conventional bracket systems was further limited by the unique V-slot design, which is characterized by minimal archwire play. The trapezoidal slot geometry is specifically intended for the leveling and alignment phase and is designed to accommodate round NiTi archwires. Rectangular archwires cannot be positioned in a standardized and reproducible manner within the trapezoidal slot and were therefore not included in the present investigation.

Another aspect to be considered was the simplified loading direction protocol, which comprised a single loading direction and one standardized malalignment configuration of 1 mm vertical offset. Previous studies have demonstrated that the degree of archwire deflection (e.g., 4 mm versus 2 mm) influenced the force release of superelastic NiTi archwires [67]. Likewise, as discussed above, increasing wire–bracket angulation has been shown to increase the contribution of binding to $R_{S}$ [38]. Consequently, both the amount of archwire deflection and bracket angulation may affect $R_{S}$. Nevertheless, the use of a standardized malalignment configuration is a well-established approach in biomechanical in vitro studies, allowing reproducible and comparable testing conditions [21,39]. Similarly, uniaxial loading is commonly employed in chewing simulator studies to ensure standardized experimental conditions [68]. While these methodological choices enhanced the reproducibility of the present study, they only partially reflect the clinical situation. During the leveling and alignment phase, archwire deflection gradually decreases as tooth alignment progresses. Therefore, the present findings are limited to the investigated malalignment configuration of 1 mm vertical offset and loading direction. Future studies should investigate multiple bracket–wire contact configurations [39] and incorporate bidirectional loading protocols [69].

No pre-vs-post experimental surface analysis was performed, as the primary aim of this study was to evaluate the functional behavior of the specific superelastic bracket wings under dynamic loading conditions. Potentially harmful preparation required for scanning electron microscopy (SEM) and for micro roughness measurements would have altered the mechanical properties of the bracket wings, thereby affecting the interpretation of the obtained results. Future studies may incorporate more specific surface characterization to investigate potential wear or surface alterations after dynamic loading.

In contrast to the clinical situation, in which teeth and their associated brackets move relative to the orthodontic archwire, the present experimental setup applied an inverted movement configuration. In mechanical and tribological testing, such an inversion of relative movement is an established approach, as tribological behavior is primarily determined by the interaction between contacting surfaces rather than by which of the two bodies is moving [70,71]. Therefore, although the direction of movement differs from the clinical situation, the fundamental tribological conditions at the bracket–archwire interface remain the same. Similar approaches using controlled relative movement conditions have been applied in orthodontic biomechanical research [72].

The findings of this biomechanical investigation should be interpreted with consideration of the limitations inherent to an in vitro experimental setup. The absence of saliva and periodontal ligament (PDL) simulation represents a simplification compared to the intraoral environment [3,40,73]. The present dry-state setup can neither fully reproduce the viscoelastic properties of the PDL nor the complex biological conditions influencing OTM, including alveolar bone remodeling, saliva, plaque accumulation, and patient-specific biological responses to mechanical loading (mechanotransduction). Therefore, the reduced frictional behavior observed under controlled experimental conditions cannot be directly translated into accelerated OTM in vivo. Nevertheless, in vitro biomechanical studies provide important insights into fundamental mechanisms underlying orthodontic appliance behavior and are essential for understanding mechanical interactions before clinical implementations [74].

A limitation of the statistical analysis was that an a priori sample size calculation could not be performed due to the novelty of the experimental design and the lack of comparable data for estimating the expected effect size. Therefore, a post hoc power analysis was conducted. However, post hoc power analyses should be interpreted with caution, as they have been shown to provide limited additional information beyond the observed statistical significance (*p*-value) [75].

Despite the limitations discussed above, the present study has several important strengths. To the authors’ knowledge, this was the first study to investigate the influence of simulated occlusal loading on resistance to sliding in a specific bracket system with superelastic bracket wings during the leveling and alignment phase. Furthermore, a novel orthodontic chewing simulator was developed, enabling controlled cyclic loading under carefully regulated temperature conditions to account for the temperature-dependent behavior of the investigated bracket as well as archwire material. The applied loading protocol incorporated clinically relevant masticatory force magnitudes, thereby providing a standardized approximation of occlusal loading under uniaxial conditions. In addition, the experimental setup was described in sufficient detail to ensure reproducibility and facilitate future investigations using comparable methodologies. The reliability of the findings was further supported by the use of complementary statistical approaches, including non-parametric analyses and linear mixed-effects modeling. Finally, the results were interpreted within the context of a comprehensive discussion of the study’s methodological limitations, allowing an appropriate assessment of their clinical relevance.

### 4.8. Clinical Implications

From a clinical point of view, the present study provides insight into the mechanical behavior of a specific orthodontic bracket–archwire system with superelastic bracket wings under functional loading conditions. Orthodontic fixed appliances are exposed not only to forces generated by the archwire itself but also to repetitive occlusal forces during mastication. These dynamic loading conditions may influence the bracket–archwire interaction, particularly during the leveling and alignment phase, when pronounced archwire deflection and close bracket–wire contact occur. The findings are particularly relevant for the leveling phase, which represents a key stage of orthodontic treatment. During this phase, initial malalignment results in pronounced archwire deflection and close bracket–archwire contact, making the mechanical behavior of superelastic bracket systems clinically relevant. The findings may be of particular relevance for any superelastic bracket system with flexible bracket components, as its mechanical behavior is specifically designed to optimize the interaction between bracket and archwire. It may be said that variations in masticatory forces between patients, may influence the response rate of OTM. The results suggest that patients with increased chewing forces (higher muscular activity) may show favorable responses in OTM if brackets with superelastic wings are used. However, whether such biomechanical effects translate into clinical conditions requires further investigation.

## 5. Conclusions

- Under the present experimental conditions, cyclic masticatory loading may reduce motion hindrances related to binding in the investigated deformable NiTi bracket system with flexible bracket wings.
- Within the limitations of the present in vitro setup, the influence of cyclic masticatory loading on archwire movement appeared to depend on the wire dimension and the applied axial activation forces. The 0.014” wire dimension demonstrated greater susceptibility to movement under low axial forces (0.4–0.6 N), whereas higher axial forces (0.8–1.0 N) were required for the 0.016” wire dimension in the investigated superelastic bracket system.
- The 0.014” NiTi wire exhibited more movement than the 0.016” NiTi wire in the specific bracket system. This difference may be related to the increased play between the bracket slot and the smaller wire dimension.
- Based on pure theoretical calculations, stainless-steel brackets were not considered for the described experiment due to the expected plastic deformation under the given experimental parameters.
- Further clinical studies are required to evaluate whether the observed effects of occlusal loading on bracket–archwire interaction translate into altered rates of tooth movement and whether they contribute to differences in orthodontic treatment duration.

## Figures and Tables

**Table 1:** Experimental parameters.

Experimental Parameter	Unit	Range
Wire diameter	Inch	0.014 and 0.016
Wire geometry	–	Round
Wire material	–	Nickel–Titanium
Motorspeed	Rpm	20
Pre-set force/chewing force	N	20–100
Displacement weight	g	43.9–105.5
Intrusion distance	mm	1
Temperature	°C	36.5 +/− 1.0
Masticatory cycles	–	250
No. of samples	–	5 to 10

**Table 2:** Both archwire dimensions with the respective applied chewing and axial forces. The range of motion for each experiment was presented as the mean, its standard deviation, median, the respective interquartile range (IQR), and the robust coefficient of variation (CV) based on IQR. A total of 5 or 10 experiments were performed in each case. For full descriptive statistics, refer to Supplementary Table S1.

Wire Dimension	Axial Force (N)	Chewing Force (N)	N	Mean (SD)	Median	IQR	Robust CV
0.014”	0.4	20	5	0.8 (0.2)	0.8	0.2	0.20
		30	5	1.2 (0.3)	1.2	0.4	0.29
		40	5	1.4 (0.3)	1.4	0.2	0.14
		70	5	1.2 (0.1)	1.2	0.2	0.17
		100	5	1.5 (0.2)	1.4	0.3	0.19
	0.6	20	10	1.9 (0.7)	1.6	1.0	0.65
		30	10	2.9 (0.9)	3.0	1.7	0.55
		40	10	2.9 (0.7)	3.0	0.8	0.26
		70	10	3.5 (1.2)	3.6	2.3	0.63
		100	10	3.5 (0.5)	3.4	0.5	0.16
	0.7	20	5	4.1 (0.6)	4.3	0.7	0.17
		30	5	4.2 (0.4)	4.1	0.2	0.04
		40	5	4.9 (0.3)	4.7	0.6	0.12
		70	5	6.4 (0.8)	6.3	0.7	0.10
		100	5	4.4 (0.7)	4.5	0.7	0.15
0.016”	0.6	20	5	0.1 (0.1)	0.0	0.1	2.14
		30	5	0.0 (0.0)	0.0	0.0	–
		40	5	0.0 (0.0)	0.0	0.0	–
		70	5	0.0 (0.0)	0.0	0.0	0.50
		100	5	0.2 (0.1)	0.1	0.1	0.47
	0.8	20	5	0.0 (0.0)	0.0	0.0	–
		30	5	0.0 (0.0)	0.0	0.0	–
		40	5	0.5 (0.3)	0.4	0.2	0.58
		70	5	0.0 (0.0)	0.0	0.0	1.00
		100	5	1.4 (0.4)	1.5	0.3	0.21
	0.9	20	10	0.1 (0.1)	0.1	0.2	2.68
		30	10	0.2 (0.3)	0.1	0.4	6.40
		40	10	0.5 (0.2)	0.5	0.2	0.47
		70	10	0.6 (0.3)	0.7	0.5	0.70
		100	10	2.6 (0.8)	2.6	0.5	0.21
	1	20	10	0.7 (0.3)	0.7	0.3	0.43
		30	10	0.9 (0.2)	1.0	0.3	0.28
		40	10	0.9 (0.1)	1.0	0.2	0.20
		70	10	1.1 (0.3)	1.0	0.2	0.21
		100	10	4.6 (0.7)	4.3	0.7	0.15

N = valid N; SD = standard deviation; IQR = interquartile range; robust coefficient of variation based on IQR.

## Data Availability

The original contributions presented in the study are included in the article/Supplementary Material; further inquiries can be directed to the corresponding authors.

## Supplementary Materials

The following supporting information can be downloaded at https://www.mdpi.com/article/10.3390/jfb17080406/s1, Table S1: Descriptive statistics of relative displacement (mm) for each wire dimension/axial force/bite force combination; Table S2: Pairwise comparisons of the chewing forces among different axial forces using the 0.014” and 0.016” NiTi archwire; Table S3: Details of post hoc power analysis using G*Power: test family ‘*t* tests’, type of power analysis: ‘Post hoc: compute achieved power’, two tails, and α probability of 0.05; Table S4: Details of the applied linear mixed model (LLM) parameters; Figure S1: Simulation of the effect of different effect sizes (d) ranging from 0.5 to 3, total sample size (5 to 100) and the achieved power; Figure S2: Estimated marginal means of relative displacement based on axial force for each level of bite force and wire dimension.
