# Supplementary material for: Influence of Mastication on Orthodontic Wire Sliding in Nickel–Titanium Brackets with Superelastic Wings
Source: J Funct Biomater. 2026 Aug 15;17(8):406. doi: 10.3390/jfb17080406 (PMC13514236; doi:10.3390/jfb17080406)
Supplement: Supplementary file 1 [file jfb-17-00406-s001.zip › jfb-4446670-supplementary.pdf]

Supplementary material for

"Influence of Mastication on Orthodontic Wire Sliding in Nickel-Titanium Brackets with Superelastic Wings"

Table S1. Descriptive statistics of relative displacement (mm) for each wire dimension / axial force / bite force combination. The range of motion for each experiment was presented as mean, its standard deviation (SD), median, the respective interquartile range (IQR) and its minimum and maximum values.

Wire dimension	Axial force (N)	Bite force (N)	n	Mean	SD[a]	Median	IQR[b]	Min.[c]	Max.[d]	Robust CV (IQR)[e]	CV[f]
0.014″	0.4	20.0	5	0.77	0.19	0.83	0.17	0.46	0.93	0.20	24.44
		30.0	5	1.16	0.27	1.25	0.36	0.81	1.47	0.29	23.46
		40.0	5	1.36	0.33	1.38	0.19	0.89	1.79	0.14	23.88
		70.0	5	1.19	0.14	1.15	0.20	1.04	1.36	0.17	11.32
		100.0	5	1.47	0.20	1.36	0.26	1.27	1.75	0.19	13.73
	0.6	20.0	10	1.87	0.70	1.62	1.05	1.05	3.20	0.65	37.47
		30.0	10	2.90	0.89	3.04	1.68	1.67	3.88	0.55	30.70
		40.0	10	2.93	0.70	3.02	0.80	1.92	3.97	0.26	23.98
		70.0	10	3.53	1.23	3.58	2.26	1.99	5.05	0.63	34.94
		100.0	10	3.50	0.46	3.41	0.54	2.76	4.32	0.16	13.05
	0.7	20.0	5	4.06	0.61	4.27	0.71	3.20	4.75	0.17	15.06
		30.0	5	4.24	0.36	4.11	0.17	3.94	4.87	0.04	8.58
		40.0	5	4.86	0.31	4.71	0.56	4.57	5.20	0.12	6.42
		70.0	5	6.42	0.82	6.32	0.66	5.37	7.58	0.10	12.72
		100.0	5	4.45	0.72	4.53	0.67	3.43	5.35	0.15	16.25
0.016″	0.6	20.0	5	0.05	0.05	0.04	0.09	-0.00	0.12	2.14	108.00
		30.0	5	0.00	0.00	0.00	0.00	0.00	0.00	Inf	100.00
		40.0	5	0.01	0.01	0.00	0.00	-0.00	0.03	Inf	216.67
		70.0	5	0.01	0.01	0.01	0.00	0.00	0.03	0.50	100.00
		100.0	5	0.18	0.13	0.15	0.07	0.09	0.42	0.47	72.83
	0.8	20.0	5	0.00	0.00	0.00	0.00	0.00	0.01	Inf	300.00
		30.0	5	0.00	0.00	0.00	0.00	0.00	0.01		Inf
		40.0	5	0.48	0.26	0.42	0.24	0.24	0.90	0.58	53.61
		70.0	5	0.00	0.00	0.00	0.00	0.00	0.00	1.00	100.00
		100.0	5	1.43	0.37	1.53	0.32	0.82	1.72	0.21	25.87
	0.9	20.0	10	0.12	0.14	0.09	0.24	-0.01	0.32	2.68	110.57
		30.0	10	0.22	0.30	0.07	0.42	0.00	0.74	6.40	132.14
		40.0	10	0.53	0.16	0.49	0.23	0.34	0.81	0.47	29.62
		70.0	10	0.64	0.32	0.67	0.47	0.21	1.18	0.70	50.55
		100.0	10	2.59	0.80	2.57	0.55	1.30	3.92	0.21	31.02
	1.0	20.0	10	0.72	0.26	0.72	0.31	0.29	1.12	0.43	36.67
		30.0	10	0.88	0.20	0.96	0.27	0.56	1.21	0.28	23.09
		40.0	10	0.95	0.13	0.97	0.20	0.76	1.13	0.20	13.52
		70.0	10	1.09	0.27	1.03	0.22	0.66	1.57	0.21	24.43
		100.0	10	4.57	0.71	4.31	0.66	3.48	6.16	0.15	15.57

[a]SD = Standard deviation; [b]IQR = Interquartile range; [c]Min. = Minimum; [d]Max. = Maximum; [e]Robust Coefficient of variation based on IQR; [f]Coefficient of variation

Table S2. Pairwise comparisons of the chewing forces among different axial forces using the 0.014″ and 0.016″ NiTi archwire. The table was divided into sections showing the applied axial forces for each archwire dimension. Lower (#1) and higher (#2) masticatory forces were compared and statistically analyzed.

Wire diameter (inch)	Axial force (N)	#1 vs #2 (N)	Difference mean (mm)	Difference SD (mm)	*P*	$P_{Adj.}$[a]	Cohen′s *d*	Power[b]
0.014	0.4	20 vs 40	-0.6	0.5	0.003	**0.035**	2.353	0.882[†]
		20 vs 100	-0.7	0.4	0.001	**0.011**	3.5	0.996[†]
	0.6	20 vs 70	-1.6	1.9	<0.001	**0.007**	1.629	0.918[†]
		20 vs 100	-1.6	1.2	<0.001	**0.002**	2.630	0.999[†]
	0.7	20 vs 70	-2.3	1.4	<0.001	**0.009**	3.252	0.991[†]
		20 vs 100	-0.3	1.3	0.439	1.000	0.460	0.096[†]
		30 vs 70	-2.2	1.2	0.001	**0.013**	3.479	0.996[†]
		30 vs 100	-0.2	1.1	0.492	1.000	0.351	0.007[†]
0.016	0.6	20 vs 100	-0.1	0.2	0.049	0.490	1	0.2723[†]
		30 vs 100	-0.2	0.1	0.001	**0.013**	2	0.9997[‡]
		40 vs 100	-0.2	0.1	0.002	**0.018**	2	0.9997[‡]
	0.8	20 vs 100	-1.4	0.4	0.002	**0.015**	3.5	1.0000[‡]
		30 vs 100	-1.4	0.4	<0.001	**0.003**	3.5	1.0000[‡]
		70 vs 100	-1.4	0.4	0.003	**0.034**	3.5	1.0000[‡]
	0.9	20 vs 70	-0.5	0.4	0.003	**0.030**	2.236	0.996[†]
		20 vs 100	-2.5	0.9	<0.001	**<0.001**	4.385	1.0000[†]
		30 vs 100	-2.4	1.1	<0.001	**<0.001**	3.973	1.0000[†]
	1.0	20 vs 100	-3.9	1.0	<0.001	**<0.001**	7.242	1.0000[†]
		30 vs 100	-3.7	0.9	<0.001	**<0.001**	7.188	1.0000[†]
		40 vs 100	-3.7	0.8	<0.001	**0.005**	7.4	1.0000[†]

*Significance values (*p*) have been adjusted by the Bonferroni correction for multiple tests. Bold letters indicate significant differences (p<0.05).

[b]Post-hoc power analysis setting: [†]Wilcoxon-Mann-Whitney U test (2 groups); [‡]Wilcoxon signed-rank test (one sample case)

Table S3. Details of post-hoc power analysis using G*Power: test famile `t tests´, type of power analysis: `Post hoc: compute achieved power´, two tails, and α probability of 0.05. Cohen's *d* was calculated using relative displacement values for the Wilcoxon-Mann-Whitney test, and mean difference of relative displacement for the Wilcoxon signed-rank test.

Wire dimension	Axial force (N)	Compared Bite force levels #1 vs #2	Relative displacement (mm)						Post-hoc power analysis		
			#1		#2		Mean difference (#1 - #2)		Statistical test	Cohen's *d*	Power
			N	Mean (SD)	N	Mean (SD)	Mean	SD			
0.014	0.4	20 vs 40	5	0.8 (0.2)	5	1.4 (0.3)	-0.6	0.5	Means: Wilcoxon-Mann-Whitney test (two groups)	2.353	0.882
0.014	0.4	20 vs 100	5	0.8 (0.2)	5	1.5 (0.2)	-0.7	0.4	Means: Wilcoxon-Mann-Whitney test (two groups)	3.5	0.996
0.014	0.6	20 vs 70	10	1.9 (0.7)	10	3.5 (1.2)	-1.6	1.9	Means: Wilcoxon-Mann-Whitney test (two groups)	1.629	0.918
0.014	0.6	20 vs 100	10	1.9 (0.7)	10	3.5 (0.5)	-1.6	1.2	Means: Wilcoxon-Mann-Whitney test (two groups)	2.630	0.999
0.014	0.7	20 vs 70	5	4.1 (0.6)	5	6.4 (0.8)	-2.3	1.4	Means: Wilcoxon-Mann-Whitney test (two groups)	3.252	0.991
0.014	0.7	20 vs 100	5	4.1 (0.6)	5	4.4 (0.7)	-0.3	1.3	Means: Wilcoxon-Mann-Whitney test (two groups)	0.460	0.096
0.014	0.7	30 vs 70	5	4.2 (0.4)	5	6.4 (0.8)	-2.2	1.2	Means: Wilcoxon-Mann-Whitney test (two groups)	3.479	0.996
0.014	0.7	30 vs 100	5	4.2 (0.4)	5	4.4 (0.7)	-0.2	1.1	Means: Wilcoxon-Mann-Whitney test (two groups)	0.351	0.007
0.016	0.6	20 vs 100	5	0.1 (0.1)	5	0.2 (0.1)	-0.1	0.2	Means: Wilcoxon-Mann-Whitney test (two groups)	1	0.2723
0.016	0.6	30 vs 100	5	0.0 (0.0)	5	0.2 (0.1)	-0.2	0.1	Wilcoxon signed-rank test (one sample case)	2	0.9997
0.016	0.6	40 vs 100	5	0.0 (0.0)	5	0.2 (0.1)	-0.2	0.1	Wilcoxon signed-rank test (one sample case)	2	0.9997
0.016	0.8	30 vs 100	5	0.0 (0.0)	5	1.4 (0.4)	-1.4	0.4	Wilcoxon signed-rank test (one sample case)	3.5	1.0000
0.016	0.8	20 vs 100	5	0.0 (0.0)	5	1.4 (0.4)	-1.4	0.4	Wilcoxon signed-rank test (one sample case)	3.5	1.0000
0.016	0.8	70 vs 100	5	0.0 (0.0)	5	1.4 (0.4)	-1.4	0.4	Wilcoxon signed-rank test (one sample case)	3.5	1.0000
0.016	0.9	20 vs 70	10	0.1 (0.1)	10	0.6 (0.3)	-0.5	0.4	Means: Wilcoxon-Mann-Whitney test (two groups)	2.236	0.996
0.016	0.9	20 vs 100	10	0.1 (0.1)	10	2.6 (0.8)	-2.5	0.9	Means: Wilcoxon-Mann-Whitney test (two groups)	4.385	1.0000
0.016	0.9	30 vs 100	10	0.2 (0.3)	10	2.6 (0.8)	-2.4	1.1	Means: Wilcoxon-Mann-Whitney test (two groups)	3.973	1.0000
0.016	1.0	20 vs 100	10	0.7 (0.3)	10	4.6 (0.7)	-3.9	1.0	Means: Wilcoxon-Mann-Whitney test (two groups)	7.242	1.0000
0.016	1.0	30 vs 100	10	0.9 (0.2)	10	4.6 (0.7)	-3.7	0.9	Means: Wilcoxon-Mann-Whitney test (two groups)	7.188	1.0000
0.016	1.0	40 vs 100	10	0.9 (0.1)	10	4.6 (0.7)	-3.7	0.8	Means: Wilcoxon-Mann-Whitney test (two groups)	7.4	1.0000

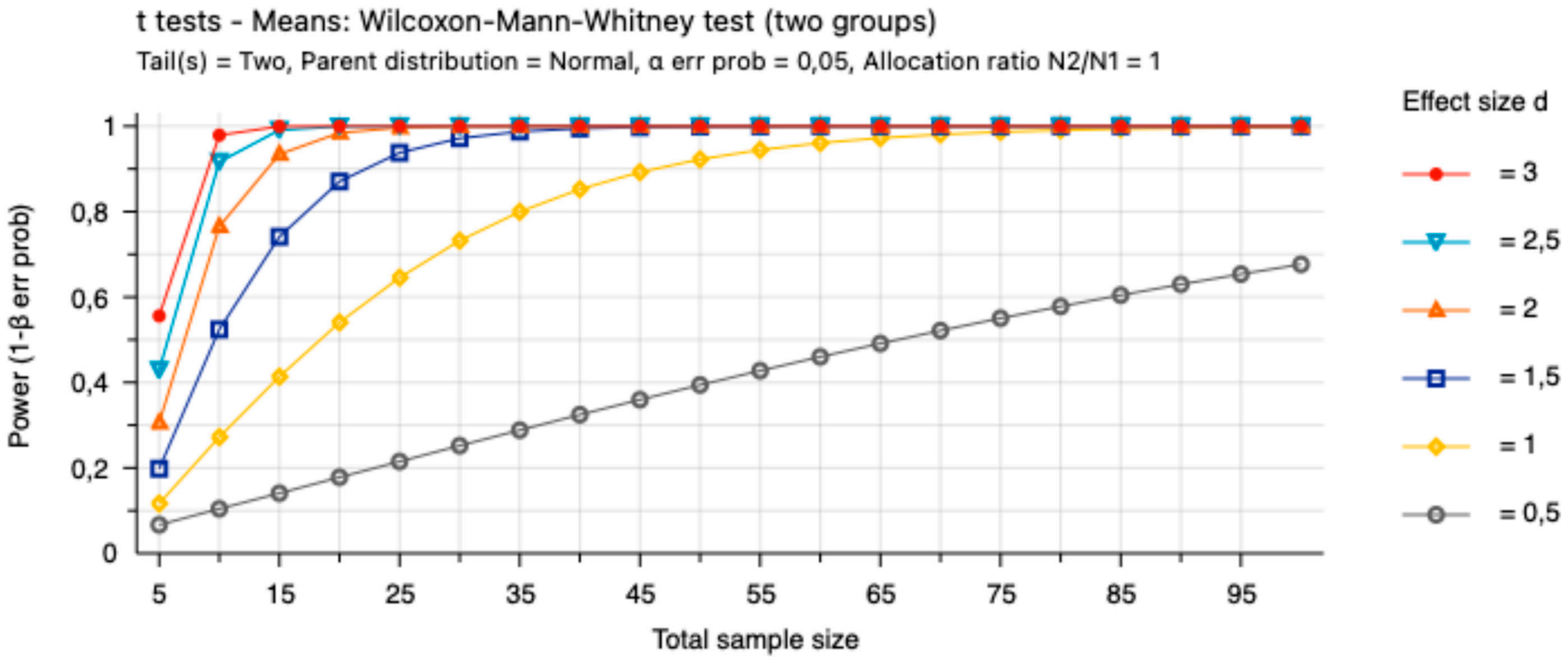

Figure S1. Simulation of the effect of different effect sizes (*d*) ranging from 0.5 to 3, total sample size (5 to 100) and the achieved power.

Table S4. Details of the applied linear mixed model (LLM) parameters.

**Predictors**		**Coefficient β**	**Coefficient (95% CI)**	***P value***
No. of wires (ID)		50		
No. of observations		250		
Fixed effects				
(Intercept)		0.48	0.07 to 0.90	**0.024**
Wire diameter				
	0.014″	*Reference*		
	0.016″	-2.90	-3.29 to -2.50	**<0.001**
Bite force:				
	20 N	*Reference*		
	30 N	0.34	-0.10 to 0.78	0.123
	40 N	0.56	0.12 to 0.99	**0.014**
	70 N	0.85	0.41 to 1.29	**<0.001**
	100 N	1.79	1.35 to 2.22	**<0.001**
Axial force:				
	0.4 N	*Reference*		
	0.6 N	1.76	1.41 to 2.10	**<0.001**
	0.7 N	3.62	3.22 to 4.02	**<0.001**
	0.8 N	2.09	1.56 to 2.62	**<0.001**
	0.9 N	2.53	2.04 to 3.01	**<0.001**
	1.0 N	3.35	2.86 to 3.83	**<0.001**
Random Effects				
Residual σ^2		0.51		
Wire ID intercept τ_{00Wire_Id}		0.13		
ICC		0.20		
Marginal R^2 / Conditional R^2		0.782 / 0.826		

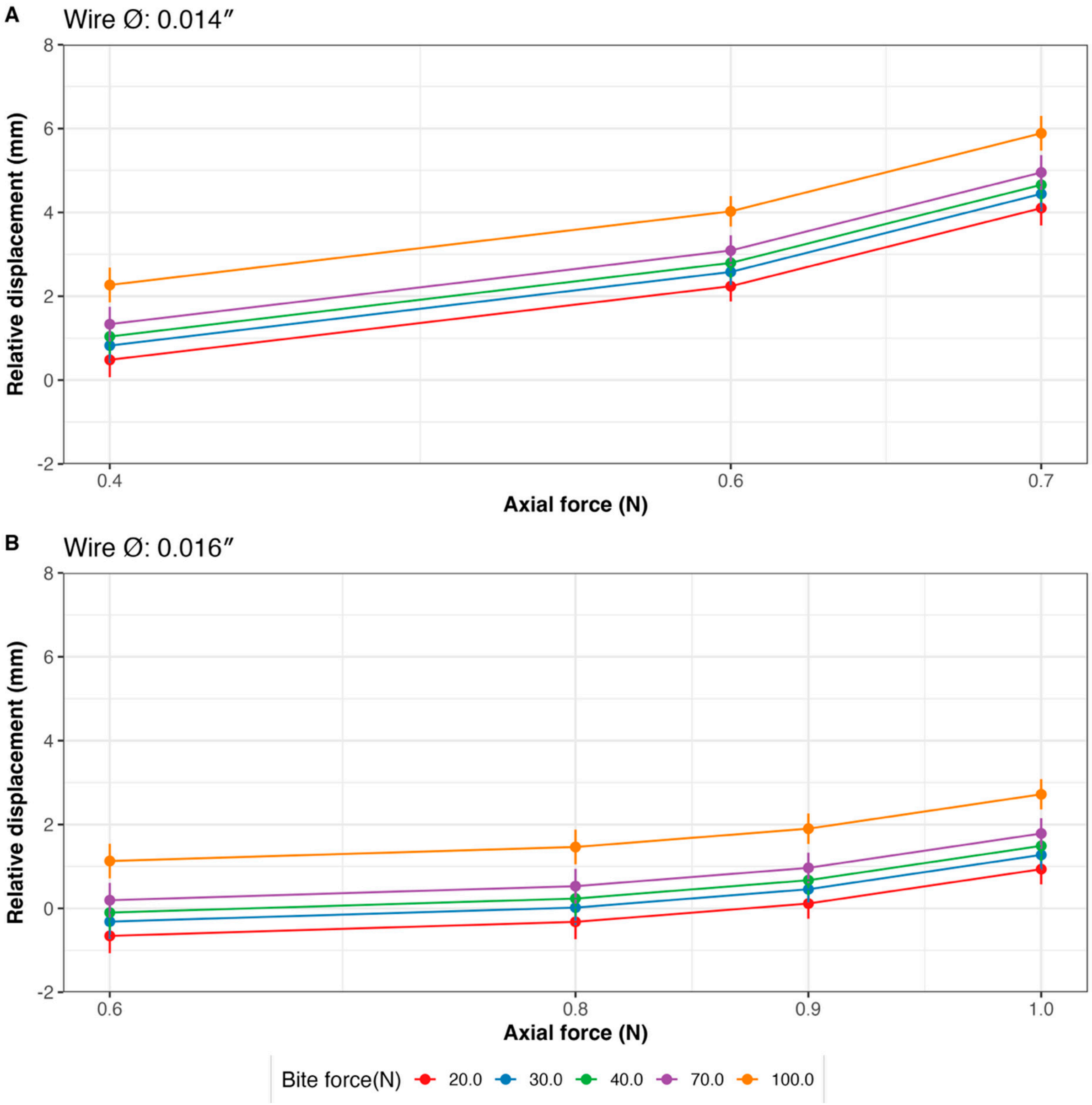

Figure S2. Estimated marginal means of relative displacement based on axial force for each level of bite force and wire dimension. Prediction is based on the fitted linear mixed model shown in Supplementary Table S4. This figure parallels Figure 4 in the manuscript.
